# Some Notes on the Mixing of Amalgams

**Published:** 1899-05

**Authors:** T. H. Dodd

**Affiliations:** Barnard Castle.


ARTICLE IV.

### SOME NOTES ON THE MIXING OF AMALGAMS.

T. H. DODD, BARNARD CASTLE.

Strange it is, but wonderfully true, that we exercise "mighty care" in having our amalgams prepared with the purest materials—the mercury distilled and redistilled to chemically purify it. Yet all this trouble is undone by the mode in which we manipulate the mixing of the metallic filling with the mercury to form a paste. Consequently many decry and condemn the alloys or mercury as not possessing (though of standard quality) the virtues they anticipate; and condemn them as inferior in quality, having failed to obtain the good results they expected; whereas often the fault rests with the operator or mixer, he having overlooked the fact that he has unconsciously, by means of the sweaty hand and bare fingers, imparted impurities that must spoil what would otherwise be an ideal filling. In referring to a recent edition of a standard work on "The Methods of Filling Teeth," etc., the publication of which has a world wide fame, I find that work falls into the same error in recommending "the mixing" to be done in a mortar, "then transferred to the palm of the hand and kneaded with the finger to amalgamate." My experience has shown me that this and other general methods adopted, using the sweaty hand, must greatly interfere with and deteriorate the quality of the amalgam so mixed, for the moisture of the hand, together with the organic acid it contains, must and will combine with the metals and mercury, and the chemical action set up, acting upon the precious metals the filling is composed of, must greatly contaminate the amalgam, rendering it somewhat valueless for the object to be effected. No doubt this accounts for the black and lusterless appearance many fillings show after a few weeks' insertion.

Let me give an explanation of what I consider will take place in amalgams prepared in the way I have described. These may be classed under three heads.

First, we will take the moisture more or less developed by the exercise of the bare finger in the bare palm of the hand. As we know, some, more than others, perspire freely. This being so, in the mixing with alloy with mercury, moisture in itself is highly prejudicial in promoting mechanical action, and a combination of the metals with the mercury. It must, more or less, stay the amalgam, preventing or arresting the plastic condition so essential to form a perfect mass.

Then we must consider the composition of the perspiration and its constituents. As I have stated, one of its elements being organic acid, will, no doubt, in turn, chemically set upon and decompose the metals of the fillings used, and this in turn forms compound salts of the metals, another impurity.

Then we have the grease contained in the perspiration —a prominent extraneous substance which will more particularly act upon the mercury, forming, I presume, "oleate of mercury." This, in like turn, must retard and arrest the mechanical combination of the metal with the mercury, reducing their affinity for each other, (the alloy and mercury), forming another impurity. Consequently there is failure in obtaining a mass that should be plastic and undiminished in permanent luster.

I would here say, failing the hand, the mortor will be best to accomplish the object of mixing, and prevent any contamination, as I have described. But experience has shown me that the device of covering the naked palm of the hand with a sheet rubber glove and the finger used for mixing with a rubber finger stall, overcomes all contamination and difficulties, and since using this I have had excellent results, obtaining a more perfect kneading, a mass retaining its fine luster, and altogether producing an ideal filling in every sense of the word.

I at first tried leather and kid, but could not get such good results as with the rubber. Another advantage rubber possesses is that it can be readily washed and quickly dried, as it becomes tarnished and soiled in the mixing process—a consideration when care has to be exercised to avoid any dampness whatever in fillings.

The squeezing of the prepared mass through a piece of chamois leather into wafers to remove excess of mercury, and "Flagg's" process of inserting a wafer or a small portion at a time into the cavity, working such portion so as to bring out the mercury to the surface, is very essential to a successful stopping.

With these few hints I close, trusting they may be of some consideration and benefit to the profession.—*The Dentist.*

